# Emergency room readmission, an avoidable problem? Analysis and stratification of readmissions in a trauma reference center

**DOI:** 10.1590/0100-6991e-20243704-en

**Published:** 2024-06-14

**Authors:** EDUARDO TAKEMURA ADANIA, GILSON SOARES DE FARIA, NICOLE RAMPANI FRANZONI, SILVANIA KLUG PIMENTEL

**Affiliations:** 1 - Hospital do trabalhador, Departamento de Cirurgia - Curitiba - PR - Brasil

**Keywords:** Accidents, Occupational. Age Groups. Emergency Medical Services. Patient Readmission. Trauma Centers, Acidentes de Trabalho, Centros de Traumatologia, Grupos Etários, Readmissão do Paciente, Serviços Médicos de Emergência

## Abstract

**Introduction:**

Hospital readmission is a common way to assess the quality of care provided in an emergency service. In this context, the aim of this study is to quantify and stratify readmissions in a trauma reference emergency service.

**Methods:**

A retrospective longitudinal study was conducted with patients readmitted, twice or more, in the emergency service within a maximum period of 30 days from the initial admission - hospitalized or not. Clinical and demographic data were obtained from electronic medical records.

**Results:**

The readmission rate for the service was 4.11% for all readmissions and 2.23% for avoidable readmissions. Within this group, 61.19% were likely avoidable, 19.47% possibly avoidable, and 19.34% eventually avoidable. Regarding time, 48.16% occurred within one week of the initial readmission. Furthermore, no statistically significant association was found in the analysis of biological sex, occupational accident, and comorbidities. A statistically significant association was found in the analysis of age and ambulance transport (OR 1.37; 95% CI 1.17-1.59).

**Conclusion:**

The study highlighted that there are still readmissions in the emergency department that could be avoided. A significant relationship was observed between readmissions and patient ages, and ambulance transport.

## RESUMO

**Introdução:**

A readmissão hospitalar é uma maneira comum de se avaliar a qualidade do atendimento prestado em um serviço de emergência. O objetivo deste estudo foi quantificar, estratificar e buscar possíveis fatores associados às readmissões de um serviço de emergência referência para atendimento ao trauma.

**Métodos:**

Estudo longitudinal retrospectivo com pacientes admitidos, duas vezes ou mais, no pronto-socorro em um período máximo de 30 dias da admissão inicial - tendo sido internados ou não. Dados clínicos e demográficos foram obtidos a partir de prontuários eletrônicos.

**Resultados:**

A taxa de readmissão do serviço foi de 4,11% para todas as readmissões e 2,23% para as readmissões evitáveis. Dentro desse grupo, 61,19% foram provavelmente evitáveis, 19,47% possivelmente evitáveis e 19,34% eventualmente evitáveis. Quanto ao tempo, 48,16% ocorreram em menos de uma semana da readmissão inicial. Além disso, não foi encontrada associação estatisticamente significativa na análise do sexo biológico, dos acidentes de trabalho e das comorbidades. Foi encontrada associação estatisticamente significativa na análise da idade e do transporte por ambulância (OR 1,37; IC 95% 1,17-1,59).

**Conclusão:**

O estudo explicitou que há readmissões em pronto-socorro que poderiam ser evitadas, além de ter sido observada uma relação significativa entre as readmissões e a faixa etária, e o transporte por ambulância.

## INTRODUCTION

The hospital readmission rate is one of the most common ways to evaluate the quality of care provided in an emergency department^1^ ^-^ ^3^. According to the National Supplementary Health Agency of the Ministry of Health, it is defined as returning to the hospital within 30 days after discharge from the first admission. However, its concept changes significantly from study to study, which undermines its usefulness to the external community^4^.

The maximum period used between initial discharge and readmission itself encourages debate in the scientific community. The 72-hour interval is the most prevalent¹ but failed to detect around 70% of readmissions^5^. Furthermore, there is a heterogeneity in the hospitals and sectors where studies are carried out - emergency rooms^6^, wards, Intensive Care Units (ICUs)^7^ - and in the patients participating in them. These associated factors reflect a variation in readmission rates from 0.07 to 33%¹.

Another important point related to readmissions is their financial consequences. Duseja et al. (2015) estimated that, in the state of Florida, the cost of readmissions was greater than 117% of the total cost of all initial admissions, including those whose patient was later readmitted. In the United States of America (USA), readmissions affect 18.2% of Medicare beneficiaries, generating a cost of 15 to 17 billion dollars^9^ ^,^ ^10^. To reduce these rates, the Hospital Readmission Reduction Program (HRRP) was established, which began to financially penalize hospital centers for higher-than-expected rates, based on performance over the previous three years.

In Brazil, publications on the topic are scarce, which makes it even more difficult to develop specific strategies for our scenario. The objective of this study was to quantify and stratify readmissions from the emergency department of a reference hospital for trauma care, in addition to characterizing the profile of the patient at greatest risk of being readmitted. 

## METHODS

This study has a retrospective, longitudinal design and is composed of patients readmitted to the emergency room from January to July 2022. The study was developed after approval by the hospital’s Ethics in Research Committee (CAAE: 63465222.7.0000.5225).

To evaluate, as a priority, the impact of the initial actions taken in the emergency room and the characteristics of the underlying cause of the first admission, we used a broader concept of readmission - an interval of 30 days between the initial admission and readmission - differently from the classic definition that delimits this period as being between initial discharge and readmission. Furthermore, we made no discrimination as to whether the patient was admitted to the service on the initial visit. The patients studied had both initial admission and readmission from January to July 2022, and we collected clinical and demographic data from electronic medical records in the service system.

We excluded cases whose first admission occurred in July and readmission in August, those whose readmission was just an accidental duplication of the first one, those with initial admission due to biological accidents, or those whose medical records from either admission were incomprehensible.

Based on the literature^11^ ^,^ ^12^, we divided readmissions into four main classifications and seven subclassifications, considering the relationship between the main complaints of emergency room admissions and the potential for readmission to be avoidable. The first major classification is “avoidable” readmission - when the complaint is closely linked to the complaint or medical management of the first admission - and was subdivided into probably, possibly, and eventually avoidable. To fit into the “probably avoidable” subgroup, readmissions must have occurred due to the persistence of the previous complaint of the initial care that was probably poorly optimized. This readmission, therefore, might not have materialized or might have been resolved in a primary health care service. For the “possibly avoidable” subgroup, readmission occurred due to possibly suboptimal first care. Finally, in the “eventually avoidable” subgroup are readmissions in which, despite the initial care having been optimized, there was some unexpected complication.

The second major classification was entitled “preference”, and it included cases in which there was withdrawal, when the patient left the emergency room before even being seen, or evasion, when he/she did so at some point before receiving medical discharge. The third category is “artifact”, cases in which either the flow of the first admission was interrupted during the initial triage and the patient was referred to primary care services or in which the patient’s readmission was, in fact, a scheduled return as per medical advice. Finally, the last category is “coincidence”, when the second admission was not associated with the first.

To descriptively explore the behavior of the data obtained, we used absolute values, frequency of the total (%), and Odds ratio (95% CI) for qualitative variables. To verify the statistical significance of the results, we used a Chi-Square model of independence, a non-parametric test, to verify the association between the categorical variables. For the relevant analyses, we performed adjusted residuals analysis.

In the first stage of the analysis, we calculated the Chi-Square value for the contingency tables, providing a general measure of the association between variables. The degrees of freedom were determined based on the number of samples and variables. The significance level chosen was α=0.05. In the end, a p-value associated with the Chi-Square <0.05 or a Chi-Square value greater than the critical value allowed rejecting the null hypothesis and favored the alternative hypothesis.

Subsequently, the analysis of adjusted residuals was performed with an alpha correction, as proposed by MacDonald and Gardner (2000), which allowed controlling the risk of type-I error when performing multiple comparisons. We calculated the adjusted residual values, p-values for each adjusted residual, and the critical Z score according to the corrected alpha. Therefore, to identify the cells that contributed significantly to the associations found, a viable approach consisted of comparing the adjusted residual values with the critical Z score and analyzing each p-value found.

All statistical analyzes and table constructions were performed using Excel, SPSS version 28.0, and Phyton statistical software.

## RESULTS

In the period from January to July 2022, 35,456 visits were made to the hospital’s emergency service by 32,489 patients, of whom 3,059 (8.63%) sought the emergency room more than once in an interval of up to 30 days. We excluded 269 visits from the study, resulting in 2,790 visits. Of these, 1,459 readmissions (Table 1) belonged to 1,331 patients. Thus, the service’s gross readmission rate was 4.11%.

**Table 1:** Stratification of readmissions.

	Readmissions	Readmission rate (n = 35,456)
Avoidable	791	2.28%
Probably	484 (61.19%)	1.38%
Possibly	154 (19.47%)	0.44%
Eventually	153 (19.34%)	0.43%
Preferences	303	0.86%
Evasion	140 (46.20%)	0.40%
Withdrawal	163 (53.80%)	0.46%
Artifacts	114	0.32%
Return request	91 (79.82%)	0.26%
Return after referral to primary services	23 (20.18%)	0.06%
Coincidences	251	0.71%
Total	1459	4.29%

Regarding the time elapsed between visits, the highest rate of readmissions occurred before the first seven days (48.17%), progressively reducing to a percentage of 11.13%, recorded after the end of the third week (Table 2).

**Table 2:** Distribution of time between the first admission and the first readmission, or between a readmission and a subsequent readmission.

Readmission time	Number of patients
< 7 days	381 (48.16%)
7-14 days	215 (27.18%)
14-21 days	107 (13.53%)
> 21 days	88 (11.13%)
Total	791

The 791 readmissions whose cause was considered avoidable are linked to 748 individuals. For statistical purposes, all analyzes from this point forward used one of the two values as a reference, depending on what was being studied, and the remainder of readmissions from the other three major classifications - “artifact”, “coincidence”, and “preference” - were considered non-readmissions.

Regarding the distribution of readmissions between men and women (Table 3), the Chi-Square test of independence showed no association between sex and a possible readmission [X²(1)=2.14; p=0.14], with a significance level of 0.05 and a critical value of 3.84.

**Table 3:** Comparative analysis between the sex of readmitted and non-readmitted patients, with a sample of 32,489 patients.

	Readmitted patients (n=748)	Patients not readmitted (n=31.741)	Odds ratio (CI 95%)
Male	427 (57.09%)	18,944 (59.68%)	0.90 (0.78 - 1.04)
Female	321 (42.91%)	12,797 (40.32%)	1.11 (0.96 - 1.29)

Regarding age groups (Table 4), the Children/Adolescents group comprised patients aged 0 to 17 years, 11 months, and 29 days (18 incomplete years), as set out in the Child and Adolescent Statute, and the Elderly group included individuals over 60 years of age, as set out in the Elderly Persons Statute. The Adults group, therefore, covered individuals outside those age groups. The Chi-Square test of independence showed an association between different age groups and patient readmission [X²(2)=69.66; p<0.001], with a significance level of 0.05 and a critical value of 5.99. Based on the analysis of the adjusted residuals (Table 4), the Children/Adolescents and Elderly group expressed significant deviations from the null expectation, as the residuals were compared with the adjusted critical value of ± 2.64 to determine which cells showed significance.

**Table 4:** Analysis of adjusted residuals for different age groups of readmitted and non-readmitted patients, with a sample of 32,489 patients.

	Readmitted (n=748)		Not readmitted (n=31.741)			
	Observed count	Expected count	Observed count	Expected count	Adjusted residuals	p-value
Age range						
Children/Adolescents	72 (9.63%)	135.7	5,836 (18.39%)	5,772.3	6.4	< 0.001
Adults	491 (65.64%)	494.3	21,024 (66.23%)	21,020.7	0.3	0.764
Elderly	185 (20.73%)	118	4,881 (15.38%)	5,016	6.8	< 0.001

Another variable covered by the study was transportation to the service by ambulance (Table 5). The Chi-Square test of independence showed an association [X²(1)=16.95; p<0.001], with a significance level of 0.05 and a critical value of 3.84.

**Table 5:** Comparative analysis between the characteristics of first admissions and care not related to readmission, with a total of 34,665 care.

	First admission (n=748)	Services not related to readmission (n=33.917)	Odds Ratio (95% CI)
Ambulance			
Yes	278 (37.17%)	10,233 (30.17%)	1.37 (1.17 - 1.59)
No	470 (62.83%)	23,684 (69.83%)	0.73 (0.63 - 0.85)
Occupational accident			
Yes	128 (17.11%)	5,550 (16.36%)	1.06 (0.87 - 1.28)
No	620 (82,89%)	28,367 (83.64%)	0.95 (0.78 - 1.15)

We also analyzed the relationship between the cause of the first admission being a occupational accident and a subsequent readmission (Table 5). The Chi-Square test of independence showed no association between occupational accidents and patient readmission [X²(1)=0.09; p=0.76], with a significance level of 0.05 and critical value of 3.84.

Finally, we investigated a possible correlation between the presence of comorbidities and the improvement, worsening, or maintenance of the severity of the clinical condition between initial admission and readmission, indicated by the Manchester Protocol classification (Table 6). In 53 cases there was no formal indication of comorbidities in the medical records, which is why they were disregarded in this analysis. In this context, the Chi-Square test of independence showed an association between the different segments of patients associated with the Manchester classification and the presence of comorbidities in patients [X²(2)=6.06; p=0.048], with a significance level of 0.05 and critical value of 5.99. However, when comparing the adjusted residuals with the adjusted critical value of ± 2.64, we found no statistically significant differences between the observed and expected frequencies, showing that all outcomes did not present significant deviations from the null hypothesis. This is because, when correcting alpha to reduce the risk of type-I (false-positive) errors, the risk of type-II (false-negative) errors can be increased.

**Table 6:** Analysis of adjusted residuals of comorbidities in relation to the Manchester classification, with a sample of 695 patients.

	With comorbidities (n=261)		No comorbidities (n=434)			
	Observed count	Expected count	Observed count	Expected count	Adjusted residuals	p-value
Classification						
Improved	99 (37.93%)	103.3	176 (40.55%)	171.7	0.7	0.484
Maintained	128 (49.04%)	132.9	226 (52.08%)	221.1	0.8	0.424
Worsened	34 (13.03%)	24.8	32 (7.37%)	41.2	2.5	0.012

## DISCUSSION

This study revealed that the gross readmission rate in the service studied was 4.11%, which is compatible with the literature¹. However, more than half (2.23%) were attributed to a preventable cause. Despite being a low percentage, the frequencies of the subgroups are different from those found in other references. In this study, the percentage of the “probably avoidable” readmission subgroup was 33.2%, while in the work by Auerbach et al. (2016) only 15% of readmissions studied presented strong or certain evidence that they could have been prevented, and in Blunt et al. (2014) the frequency was 5.39%. These discrepancies may be due to the circumstances of the major cases, in this case, emergency trauma in a public service with high demand. The lack of adequate post-discharge guidance from the medical team is certainly a hypothesis to be raised, as there were many cases in which patients returned to the service with the same pain complaint and no new findings were found, maintaining the previous conduct. Furthermore, the academic profile of the service may have contributed to unsatisfactory assistance.

However, the other major classifications of readmissions are not considered inert and uninterventionable. The “preference” category, when patients interrupt care by their own decision and then return, were 20.77% of the service’s readmissions, and part of them occurred due to the delay in care upon initial admission, as described in the medical records. And in the “artifact” category, 20.18% represent patients who returned after initial referral to a less complex service, denoting a lack of coordination in the care network and low resolution in the situation of mild trauma on the part of primary care services, which can be considered a contributing factor to the overload of the emergency service and consequent long waiting time for care.

Regarding the time elapsed between initial admission and readmission, it was less than a week in around 50% of cases, a finding quite like that found by Considine et al. (2017). Rising et al. (2014) proposed an interval of nine days as ideal, a cutoff that would render approximately 40% of readmissions unnoticed in our series. Another hypothesis to be put forward is that readmissions in less than seven days would be related to the “probably avoidable” subclassification, since, for the most part, they were either caused by poor guidance or a gross misdiagnosis.

Male sex was the most prevalent in general, and we found no statistically significant association between biological sex and readmission, which is in line with the results of other studies^1^ ^,^ ^14^. What probably explains this disparity between sexes is the higher prevalence of men in cases involving trauma^15^ - a situation with higher prevalence in this emergency service -, since we also observed the same proportionality in the non-readmitted group of patients.

Regarding age, while the frequency of adults found in the groups of readmitted and non-readmitted patients remained the same, the prevalence of the elderly population increased by almost 35% in the first group. One of the possible causes for this increase, also found in the literature^16^, is the decrease in physiological reserves in the elderly^17^, which makes them more vulnerable to acute stress and slows recovery from injuries. On the other hand, the pediatric population showed a significant reduction in the readmitted group, an important finding of this study, as this group of individuals is usually excluded from the analysis of research involving general readmissions.

Another relevant point of this study is the possibility of predicting a group that is at greater risk of being readmitted before their care even begins. Although the study is retrospective and makes it difficult to infer a risk correlation, the patient’s arrival by ambulance on their first visit to the service was more associated with future readmission (OR 1.37). Chan et al. (2020), when faced with the same association, developed the hypothesis that the greatest activation of the ambulance protocol occurred in long-stay institutions for the elderly, and due to the inability to deal with the patient’s symptoms, they soon return to the hospital Emergency Room. However, this theory does not include the pediatric and adult population. Another plausible explanation for the finding is the greater complexity/severity of cases when transport by an emergency care unit occurs^18^, which is a factor more associated with readmissions^19^.

As for occupational accidents, there was a high prevalence of emergency room visits, as expected^20^. The frequencies of such injuries in the group of readmitted and non-readmitted patients were similar to each other, justifying the association between occupational accidents and readmission not being statistically significant. We found no studies that addressed a relationship between occupational accidents and readmissions, which reinforces the need for more research in this regard.

Finally, we evaluated the presence of comorbidities worsening the patient’s condition upon readmission. Comorbidities are associated with readmission²¹, and therefore we expected a worsening of clinical evolution. Initially, we found a slight difference between the expected and observed counts, but in the comparison test, carried out to avoid type-I errors, proved to be statistically insignificant.

Limitations of the study were the omission of information, discrepancy between evolutions, and part of the visits occurring in the first quarter of 2022, during the COVID-19 pandemic, which may have changed the flow of patients.

## CONCLUSION

Although the readmission rate was within expectations, 4.11%, more than half of cases could have been avoided. Furthermore, elderly patients and those transported by ambulance had a greater chance of being readmitted. Such findings are of great value to the epidemiological sector and service coordination, as they make it possible to list hypotheses regarding readmissions, to assist in planning actions to make care for the population more effective, also reducing costs and other social charges.
